# Insights into phylogenetic positions and distribution patterns: Complete mitogenomes of two sympatric Asian horned toads in *Boulenophrys* (Anura: Megophryidae)

**DOI:** 10.1002/ece3.11687

**Published:** 2024-07-10

**Authors:** Hongmei Xiang, Qiang Zhou, Wei Li, Juan Shu, Zhirong Gu, Wansheng Jiang

**Affiliations:** ^1^ Key Laboratory of Hunan Forest Products and Chemical Industry Engineering, National and Local United Engineering Laboratory of Integrative Utilization Technology of Eucommia ulmoides Jishou University Zhangjiajie China; ^2^ College of Biology and Environmental Sciences Jishou University Jishou China; ^3^ Zhangjiajie National Forest Park Zhangjiajie China; ^4^ National Nature Reserve of Badagongshan Zhangjiajie China

**Keywords:** Asian horned toad, China, Megophryidae, mitochondrial genome, phylogenetic analysis

## Abstract

*Boulenophrys sangzhiensis* and *Boulenophrys tuberogranulata*, two narrow‐distributed toad species within the Megophryidae family in southern China, are experiencing population declines due to habitat loss and degradation. Despite their critical conservation status, the two species remain largely overlooked in public and scientific spheres. This study presented the first sequencing, assembly, and annotation of the complete mitogenomes of both species using next‐generation sequencing. The mitogenome of *B. sangzhiensis* was 16,950 bp, while that of *B. tuberogranulata* was 16,841 bp, each comprising 13 protein‐coding genes (PCGs), 22 transfer RNA genes (tRNAs), two ribosomal RNA genes (rRNAs), and a noncoding control region (D‐loop). The gene content, nucleotide composition, and evolutionary rates of each mitogenome were analyzed. Both mitogenomes exhibited negative AT skew and GC skew with high A + T content. ATP8 exhibited the highest evolutionary rate, while COI had the lowest. A phylogenetic analysis based on 28 mitogenomes revealed two major clades of Megophryidae, supporting the classification of two subfamilies, Megophryinae and Leptobrachiinae. Within the subfamily Megophryinae, the genus *Boulenophrys* was divided into two species groups. Intriguingly, despite coexisting in Zhangjiajie City, *B. sangzhiensis* and *B. tuberogranulata* exhibited distinct origins from the two different species groups, underscoring the unique role of the coexisting area Zhangjiajie in driving their speciation and preserving their current populations. A parallel pattern was also identified in the Leptobrachiinae genus *Leptobrachium* within the same region. This study provided valuable data references and enhanced our understanding of the molecular characteristics of these threatened amphibian species.

## Graphical abstract

The mitogenomes of two endemic and threatened Asian horned toads, *Boulenophrys sangzhiensis* and *Boulenophrys tuberogranulata*, were sequenced and reported for the first time. A phylogenetic reconstruction based on all available mitogenomes within Megophryidae revealed a very special phylogenetic and distributional pattern of the two sympatric species relative to other congeners in *Boulenophrys*.

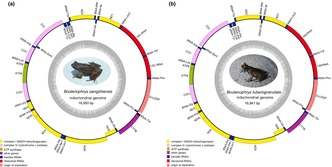

## 1 INTRODUCTION

The toad family Megophryidae (Bonaparte, 1850) is an Asian endemic group that primarily distributed from eastern Pakistan to western China, and southward to the Philippines and the Greater Sunda Islands (Frost, 2023). Although the taxonomic validity of Megophryidae has been challenged for a long time in the last century (Zhou et al., 2023), it is now generally recognized as a monophyletic group and includes two subfamilies, Megophryinae and Leptobrachiinae (AmphibiaWeb, 2023; Frost, 2023). According to the latest statistics, the two subfamilies comprise 134 and 187 species, respectively (Frost, 2023), making Megophryidae one of the most diverse groups of amphibians in Asia as well as in the world.

The subfamily Megophryinae (Bonaparte, 1850), also known as Asian horned (or spadefoot) toads, basically reflects the distribution range and pattern of the whole Megophryidae family (Frost, 2023). Due to significant morphological diversity and similarity, especially the lack of easily recognizable generic diagnostic characters, debates regarding generic classifications within Megophryinae have persisted for decades. Several different generic hypotheses have been proposed from various studies, including five‐genus classifications (Chen et al., 2017; Delorme et al., 2006), seven‐genus classifications (Dubois et al., 2021; Fei & Ye, 2016; Lyu et al., 2021; Qi et al., 2021), or a single genus proposal encompassing all Megophryinae species together as a large group (Mahony et al., 2017). These specific generic classifications have attracted dedicated followings in later studies, being embraced or revised by databases over time. Among them, the proposal of a single large genus by Mahony et al. (2017) has been widely accepted in various studies (e.g., Gao et al., 2022; Liu et al., 2018; Tapley et al., 2021; Wang et al., 2019, 2020). However, a recent study has introduced a 10‐genus classification from an integrative taxonomic perspective (Lyu et al., 2023). This new classification is revealed by 10 well‐supported molecular phylogenetic clades and presented with combined morphological diagnoses for each genus, representing the most reliable and the latest taxonomic recognition of Megophryinae. These genera include *Atympanophrys*, *Boulenophrys*, *Brachytarsophrys*, *Grillitschia*, *Jingophrys*, *Megophrys*, *Ophryophryne*, *Pelobatrachus*, *Sarawakiphrys*, and *Xenophrys*. This 10‐genus classification of Megophryinae has been adopted shortly in mainstream databases such as Amphibian Species of the World (https://amphibiansoftheworld.amnh.org/) and AmphibiaChina (https://www.amphibiachina.org/), which is also the updated taxonomic system we used in this study.

**FIGURE 1:**
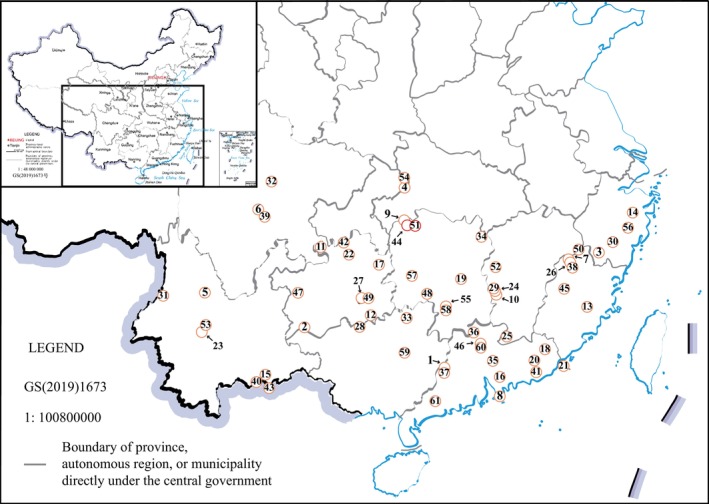
The recorded distribution sites of *Boulenophrys* species in China (data from AmphibiaChina, 2024). The circles (in red show the species we collected in this study) on the map represent the type localities of *Boulenophrys* species distributed in China or near the national boundaries. The numbers 1–61 represent the species name as follow: (1) *B. acuta*, (2) *B. anlongensis*, (3) *B. baishanzuensis*, (4) *B. baolongensis*, (5) *B. binchuanensis*, (6) *B. binlingensis*, (7) *B. boettgeri*, (8) *B. brachykolos*, (9) *B. caudoprocta*, (10) *B. cheni*, (11) *B. chishuiensis*, (12) *B. congjiangensis*, (13) *B. daiyunensis*, (14) *B. daoji*, (15) *B. daweimontis*, (16) *B. dongguanensis*, (17) *B. fanjingmontis*, (18) *B. fengshunensis*, (19) *B. hengshanensis*, (20) *B. hungtai*, (21) *B. insularis*, (22) *B. jiangi*, (23) *B. jingdongensis*, (24) *B. jinggangensis*, (25) *B. jiulianensis*, (26) *B. kuatunensis*, (27) *B. leishanensis*, (28) *B. liboensis*, (29) *B. lini*, (30) *B. lishuiensis*, (31) *B. lushuiensis*, (32) *B. minor*, (33) *B. mirabilis*, (34) *B. mufumontana*, (35) *B. nankunensis*, (36) *B. nanlingensis*, (37) *B. obesa*, (38) *B. ombrophila*, (39) *B. omeimontis*, (40) *B. palpebralespinosa*, (41) *B. puningensis*, (42) *B. qianbeiensis*, (43) *B. rubrimera*, (44) *B. sangzhiensis*, (45) *B. sanmingensis*, (46) *B. shimentaina*, (47) *B. shuichengensis*, (48) *B. shunhuangensis*, (49) *B. spinata*, (50) *B. tongboensis*, (51) *B. tuberogranulata*, (52) *B. wugongensis*, (53) *B. wuliangshanensis*, (54) *B. wushanensis*, (55) *B. xiangnanensis*, (56) *B. xianjuensis*, (57) *B. xuefengmontis*, (58) *B. yangmingensis*, (59) *B. yaoshanensis*, (60) *B. yingdeensis*, (61) *B. yunkaiensis*.

The genus *Boulenophrys* (Fei & Ye, 2016), which includes half of the recognized species within Megophryinae (67 out of 134 total species), is the largest genus in Megophryinae (Frost, 2023). China hosts the most diverse population of Megophryinae, housing over two‐thirds of the total cataloged species (Lyu et al., 2023). Unsurprisingly, species in *Boulenophrys* are predominantly found in southern China (61 species, see Figure 1), with a few extension westwards into Myanmar and southwards into northernmost Indochina, including Vietnam, Laos, and Thailand (Lyu et al., 2023). Navigating the intricate taxonomic history of Megophryinae, *Boulenophrys* has undergone several revisions despite being a relatively recent‐established genus (Fei & Ye, 2016). It was frequently considered as a subgenus or synonymous of *Megophrys*, *Panophrys*, or *Xenophrys* (e.g., Chen et al., 2017; Luo et al., 2021; Lyu et al., 2021; Mahony et al., 2017) until Dubois et al. (2021) recognized that the name *Panophrys* was preoccupied. They then reestablished *Boulenophrys* as the valid generic name following the International Code of Zoological Nomenclature. This recognition has been subsequently adopted by other studies (Lyu et al., 2023; Qi et al., 2021; Wang, Zeng et al., 2022) and databases (AmphibiaWeb, 2023; Frost, 2023).

Majority of species within *Boulenophrys* exhibit narrow distributions and small populations. For instance, *Boulenophrys sangzhiensis* (Jiang et al., 2008) is exclusively known from Mt. Tianping and Mt. Huping in northwestern Hunan Province, lived at elevations of 1300–1400 m a.s.l. (Fei & Ye, 2016; Lyu et al., 2023). Similarly, *Boulenophrys tuberogranulata* (Mo et al., 2010) is restricted to Tianzishan Nature Reserve and Mt. Tianping in northwestern Hunan Province, found at elevations ranging from 1000 to 1380 m a.s.l. (Lyu et al., 2023; Mo et al., 2010). Notably, *B. sangzhiensis* and *B. tuberogranulata* share overlapping distributions in Mt. Tianping. Despite this, they exhibit distinct morphological characteristics, suggesting a potentially early derivation from respective evolutionary lineages. Both species, however, face population declines due to threats such as habitat loss or degradation (Gao et al., 2022). The IUCN SSC Amphibian Specialist Group assessed their status in 2020, classifying *B. sangzhiensis* as Critically Endangered (CR) and *B. tuberogranulata* as Endangered (EN) on the IUCN Red List of Threatened Species (IUCN SSC Amphibian Specialist Group, 2020a, 2020b).

As endemic yet threatened amphibians, both *B. sangzhiensis* and *B. tuberogranulata* have received very limited public attentions and scientific concerns. Knowledges about these species in science have primarily been limited to initial morphological descriptions and distributions, with little engagement in phylogenetic studies, let alone investigations of their population structure and genetic diversity. Moreover, given the existence of over 60 species in *Boulenophrys*, constructing a robust phylogenetic tree using mitogenomes, for example, could significantly enhance our understanding of the phylogeny, trait evolution, and current distribution patterns within this group. However, the available mitogenome data remain quite limited. In this study, we utilized next‐generation sequencing (NGS) technology to sequence and assemble the complete mitogenomes of *B. sangzhiensis* and *B. tuberogranulata* for the first time. Our primary aim was to conduct a detailed analysis and description of these two complete mitogenomes to enhance the mitogenomic data of *Boulenophrys*. Then, we reconstructed a phylogenetic tree of Megophryidae based on all available mitogenomes in GenBank to scrutinize the current understanding of the phylogeny of this group. This study is expected to provide valuable data references for population genetics and conservation biology studies on these two threatened species. It will also contribute to the phylogenetic knowledge of *Boulenophrys* and other Asian horned toads in the future.

## 2 MATERIALS AND METHODS

### 2.1 Sample collection and sequencing

Permissions for the field survey in this study were obtained for scientific purposes from the local administrations, and the sample collections and experiment protocols were approved by the Biomedical Ethics Committee of Jishou University (No: JSDX‐2024‐0083) adhered to the relevant laws and guidelines of China. Following the “3R principle” (Reduction, Replacement, and Refinement) of animal ethics that required by National Ministry of Science and Technology (No. 398 [2006]), only one sample of each species was utilized. The sample of *B. sangzhiensis* was collected in May 2021 from its type locality within the National Nature Reserve of Badagongshan in Sangzhi County. The sample of *B. tuberogranulata* was collected in August 2022, also from its type locality within the Zhangjiajie National Forest Park in Wulingyuan District. The straight‐line distance between the two sample localities was only 60 km, and both are situated in Zhangjiajie City, Hunan Province, China. All animal collection and treatment procedures strictly followed the guidance outlined in the Code of Practice for the Housing and Care of Animals. The specimens were euthanized and preserved in 85% alcohol as voucher specimens, and then deposited at the molecular ecology laboratory in Zhangjiajie Campus, Jishou University (*B. sangzhiensis*, voucher no. JWS20210007; *B. tuberogranulata*, voucher no. JWS20221040). A small section of liver tissue from each sample was used for molecular analysis. Total DNA extraction was performed using the DNeasy Blood & Tissue Kit (Qiagen), and the DNA library was constructed using the VAHTS Universal DNA Library Prep Kit for Illumina V3 (Vazyme). High‐throughput sequencing was conducted in paired‐end mode on the DNBSEQ‐T7 platform (Complete Genomics and MGI Tech), generating approximately 30 Gb of raw reads with a read length of 150 bp for each sample.

### 2.2 Sequence assembly, annotation, and analysis

The complete mitogenomes of *B. sangzhiensis* and *B. tuberogranulata* were assembled using NOVOPlasty 4.3 (Dierckxsens et al., 2016), with the mitogenome of *Boulenophrys jingganggensis* serving as a reference. The assembled sequences were then queried against the standard database of NCBI using the BLAST online program to identify highly similar sequences. The positions and directions of protein‐coding genes (PCGs), ribosomal RNA genes (rRNAs), transfer RNA genes (tRNAs), and the control region (D‐loop) were annotated using the MITOS Web server (Bernt et al., 2013). This annotation was cross‐verified by comparing it with the information available for congeners. Further annotation and gene map visualization were carried out using the web application GeSeq (Tillich et al., 2017). Codon usage and nucleotide frequencies of the PCGs were determined using MEGA X (Kumar et al., 2018). AT skew and GC skew were calculated using the formulas: AT skew = (A – T)/(A + T) and GC skew = (G – C)/(G + C) (Perna & Kocher, 1995). The relative synonymous codon usage (RSCU), nonsynonymous substitution rate (Ka), and synonymous substitution rate (Ks) of all PCGs were analyzed using DnaSP V6 (Rozas et al., 2017) to investigate potential signals of selective pressure.

### 2.3 Phylogenetic analysis

All available mitogenomes of Megophryidae species from GenBank were downloaded, and one representative sequence was selected for each species. A comprehensive dataset comprising a total of 28 Megophryidae species, including the newly sequenced *B. sangzhiensis* and *B. tuberogranulata*, was compiled for phylogenetic reconstruction. As an outgroup, *Microhyla fissipes* from the family Microhylidae was included. PhyloSuite (Zhang et al., 2020) was employed to extract each of the 13 PCGs from the 29 mitogenomes. Subsequently, individual PCG datasets were aligned using the MUSCLE module in MEGA X and manually checked for accuracy. Finally, these aligned and trimmed datasets were concatenated to create a combined PCGs dataset using PhyloSuite. Phylogenetic trees were reconstructed using both the maximum likelihood (ML) method with RAxML 8.0.2 (Stamatakis, 2006) and Bayesian inference (BI) method using MrBayes 3.2.7 (Ronquist & Huelsenbeck, 2003). The best‐fit partitioning scheme and nucleotide substitution models for ML and BI reconstructions were determined using PartitionFinder 2 (Lanfear et al., 2017). Statistical confidence was assessed by conducting a bootstrap test with 1000 replicates for ML trees. For BI trees, posterior probabilities were calculated under a simultaneous run of 1.0 × 10^7^ million generations, with sampling every 1000 generations and discarding the initial 25% of generations as burn‐in.

## 3 RESULTS

### 3.1 Mitogenome assembly, annotation, and nucleotide composition

**FIGURE 2:**
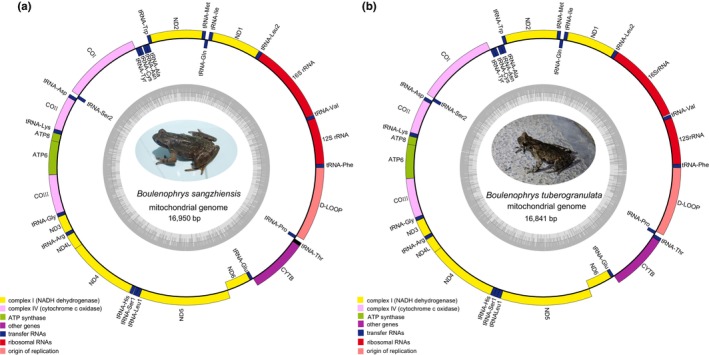
Gene map of the mitogenome of *Boulenophrys sangzhiensis* (a) and *Boulenophrys tuberogranulata* (b).

The complete mitogenome of *B. sangzhiensis* was 16,950 bp, while that of *B. tuberogranulata* was 16,841 bp in length after assembly using NOVOPlasty (Figure 2). Blastn searches revealed that the most similar sequences in NCBI to be those from *Boulenophrys* and the best‐matched sequences showed the species identification was correct. Both mitogenomes exhibited the typical characteristics of animal mitogenomes, comprising 37 genes, including 13 PCGs, 22 tRNAs, two rRNAs, and a D‐loop control region (Table 1 and Figure 2). Most of these genes were encoded by the heavy strand (H‐strand), except for one PCG (ND6) and eight tRNAs, which were encoded on the light strand (L‐strand). The final complete mitogenomes, along with annotated information for both species, have been deposited in GenBank under accession numbers OQ830572 and OQ830573.

**TABLE 1:** Characteristics of the mitogenome of *Boulenophrys sangzhiensis* (BS) and *Boulenophrys tuberogranulata* (BT).

Genes	Position		Length		Start codon		Stop codon		Anticodon	Strand	Intergenic nucleotide	
	BS	BT	BS	BT	BS	BT	BS	BT	BS/BT	BS/BT	BS	BT
tRNA^Phe^	1–70	1–71	70	71					GAA	H	0	0
12S	71–995	72–996	925	925						H	–4	–4
tRNA^Val^	992–1061	993–1063	70	71					TAC	H	3	14
16S	1065–2658	1078–2671	1594	1594						H	–2	–2
tRNA^Leu2^	2657–2731	2670–2743	75	74					TAA	H	0	0
ND1	2732–3705	2744–3717	974	974	ATA	ATG	TA(A)	TA(A)		H	3	3
tRNA^Ile^	3709–3779	3721–3791	71	71					GAT	H	−1	–1
tRNA^Gln^	3779–3850	3791–3862	72	72					TTG	L	–1	–1
tRNA^Met^	3850–3918	3862–3930	69	69					CAT	H	0	0
ND2	3919–4959	3931–4971	1041	1041	ATG	ATT	TAG	TAG		H	−2	−2
tRNA^Trp^	4958–5026	4970–5038	69	69					TCA	H	0	0
tRNA^Ala^	5027–5095	5039–5107	69	69					TGC	L	0	0
tRNA^Asn^	5096–5168	5108–5180	73	73					GTT	L	2	2
OL	5171–5198	5183–5212	28	30						H	−1	−1
tRNA^Cys^	5198–5260	5212–5274	63	63					GCA	L	0	0
tRNA^Tyr^	5261–5327	5275–5341	67	67					GTA	L	1	1
COI	5329–6885	5343–6905	1557	1563	TTG	GTG	AGA	AGA		H	–5	−10
tRNA^Ser2^	6881–6951	6896–6966	71	71					TGA	L	3	3
tRNA^Asp^	6955–7021	6970–7037	67	68					GTC	H	0	0
COII	7022–7706	7038–7722	685	685	ATG	ATG	T(AA)	T(AA)		H	0	0
tRNA^Lys^	7707–7779	7723–7795	73	73					TTT	H	1	1
ATP8	7781–7945	7797–7961	165	165	ATG	ATG	TAA	TAA		H	−10	−10
ATP6	7936–8618	7952–8634	683	683	ATG	ATG	TA(A)	TA(A)		H	−1	−1
COIII	8618–9402	8634–9418	785	785	ATG	ATG	TA(A)	TA(A)		H	−1	−1
tRNA^Gly^	9402–9470	9418–9486	69	69					TCC	H	0	0
ND3	9471–9815	9487–9831	345	345	TTG	ATG	TAG	TAG		H	−2	−2
tRNA^Arg^	9814–9882	9830–9898	69	69					TCG	H	3	3
ND4L	9886–10,182	9902–10,198	297	297	ATG	ATG	TAA	TAA		H	−7	−7
ND4	10,176–11,553	10,192–11,572	1378	1381	ATG	ATG	T(AA)	T(AA)		H	6	0
tRNA^His^	11,560–11,628	11,573–11,641	69	69					GTG	H	6	0
tRNA^Ser1^	11,635–11,696	11,642–11,708	62	67					GCT	H	0	0
tRNA^Leu1^	11,697–11,769	11,709–11,781	73	73					TAG	H	0	0
ND5	11,770–13,587	11,782–13,599	1818	1818	ATG	ATG	TAA	TAA		H	7	3
ND6	13,595–14,104	13,603–14,112	510	510	ATG	ATG	AGG	AGA		L	0	0
tRNA^Glu^	14,105–14,173	14,113–14,181	69	69					TTC	L	2	2
Cytb	14,176–15,315	14,184–15,323	1140	1140	ATG	ATG	TAG	TAG		H	–1	–1
tRNA^Thr^	15,315–15,385	15,323–15,392	71	70					TGT	H	0	1
tRNA^Pro^	15,386–15,454	15,394–15,462	69	69					TGG	L	0	–1
D‐loop	15,455–16,950	15,462–16,841	1496	1380						H	0	0

A total of 38 and 44 bp overlapping sites were shared in 13 and 14 pairs of neighboring genes in *B. sangzhiensis* and *B. tuberogranulata*, respectively, with lengths ranging from 1 to 10 bp. Additionally, there were 37 and 32‐bp intergenic nucleotides (IGNs) dispersed across 11 and 10 locations in the two species, with lengths ranging from 1 to 7 bp and 1–14 bp, respectively. Both species featured a short noncoding region located between tRNA^Asn^ and tRNA^Cys^, measuring 28 bp in *B. sangzhiensis* and 30 bp in *B. tuberogranulata*. The nucleotide compositions of *B. sangzhiensis* were 27.45% A, 30.77% T, 14.81% G, and 26.97% C, while those of *B. tuberogranulata* were 27.79% A, 30.91% T, 14.55% G, and 26.75% C (Table 2). Similar to other species in Megophryidae, all the A + T values accounted for more than half, indicating an A + T bias with greater A + T than G + C contents. For the H‐strand sequence of all examined Megophryidae species, both AT skew and GC skew were negative, indicating a predominant bias toward T and C base pairs.

**TABLE 2:** Base composition (in percentages) of the mitogenomes of *Boulenophrys sangzhiensis*, *Boulenophrys tuberogranulata,* and other 26 species in Megophryidae.

Species	Total length	T(U)%	C%	A%	G%	A + T%	AT skew	GC skew	Accession numbers
*Boulenophrys sangzhiensis*	16,950	30.77	26.97	27.45	14.81	58.21	−0.057	−0.291	OQ830572 ^a^
*Boulenophrys tuberogranulata*	16,841	30.91	26.75	27.79	14.55	58.70	−0.053	−0.295	OQ830573 ^a^
*Boulenophrys jingganggensis*	17,262	31.62	26.28	27.62	14.48	59.24	−0.067	−0.289	MT683772
*Boulenophrys omeimontis*	17,013	31.76	25.72	28.34	14.18	60.10	−0.057	−0.289	KP728257
*Boulenophrys spinata*	16,024	30.63	27.11	27.16	15.10	57.79	−0.060	−0.284	ON646614
*Boulenophrys boettgeri*	16,597	31.51	26.42	27.84	14.23	59.35	−0.062	−0.300	OR529440
*Boulenophrys kuatunensis*	17,921	32.07	25.86	28.10	13.97	60.17	−0.066	−0.298	OR522721
*Boulenophrys baishanzuensis*	17,040	31.51	26.64	27.24	14.61	58.75	−0.073	−0.292	OR063945
*Brachytarsophrys carinense*	15,271	29.80	27.61	27.46	15.13	57.26	−0.041	−0.292	JX564854
*Leptobrachium boringii*	17,097	31.53	25.51	27.69	15.27	59.22	−0.065	−0.251	OP373724
*Leptobrachium liui*	17,499	32.74	24.32	28.10	14.84	60.84	−0.076	−0.242	OP503540
*Oreolalax major*	17,786	32.63	24.31	28.75	14.31	61.37	−0.063	−0.259	MN803320
*Oreolalax xiangchengensis*	17,110	33.02	23.62	29.18	14.18	62.20	−0.062	−0.250	MH727696
*Oreolalax jingdongensis*	17,864	32.73	23.92	29.10	14.26	61.82	−0.059	−0.253	MF953479
*Oreolalax omeimontis*	17,675	32.59	24.96	28.46	13.99	61.05	−0.068	−0.282	MN803321
*Oreolalax multipunctatus*	17,358	32.96	24.20	28.53	14.31	61.49	−0.072	−0.257	MF966382
*Oreolalax lichuanensis*	17,702	32.17	24.86	28.00	14.98	60.17	−0.069	−0.248	KU096847
*Oreolalax schmidti*	18,481	32.80	24.48	28.31	14.41	61.11	−0.073	−0.259	MT773151
*Oreolalax rhodostigmatus*	18,676	32.39	24.93	28.03	14.66	60.42	−0.072	−0.259	MF770485
*Leptobrachium ailaonicum*	17,318	31.83	24.99	27.90	15.27	59.74	−0.066	−0.241	MZ394043
*Leptobrachium leishanense*	17,485	32.64	24.38	28.15	14.83	60.79	−0.074	−0.244	KU760082
*Scutiger ningshanensis*	17,265	32.68	24.25	29.11	13.96	61.79	−0.058	−0.269	KX619450
*Scutiger liupanensis*	16,888	32.25	24.90	28.32	14.53	60.57	−0.065	−0.263	KX352261
*Leptolalax oshanensis*	17,747	29.85	26.26	28.77	15.11	58.62	−0.018	−0.270	KC460337
*Leptobrachella alpina*	17,763	30.77	25.64	28.53	15.05	59.30	−0.038	−0.260	MW487804
*Leptolalax pelodytoides*	14,682	29.07	27.80	27.67	15.46	56.74	−0.025	−0.285	JX564874
*Atympanophrys shapingensis*	17,631	31.48	26.05	28.18	14.29	59.66	−0.055	−0.291	JX458090
*Atympanophrys gigantica*	18,259	32.11	25.19	28.37	14.33	60.48	−0.062	−0.275	MZ364157
*Microhyla fissipes*	16,723	31.01	25.48	28.93	14.58	59.94	−0.035	−0.272	MN046210

^a^ The sequence obtained in this study.

### 3.2 Characteristics of PCGs and codon usage

The total length of the PCGs in *B. sangzhiensis* was 11,378 bp, and in *B. tuberogranulata*, it was 11,387 bp. The differences in length were attributed to 6 bp in COI and 3 bp in ND4, which were longer in *B. tuberogranulata* than in *B. sangzhiensis*; the lengths of other PCGs were identical in both species. Most PCGs in both species commenced with the ATG codon, except for ND1 using ATA, COI, and ND3 using TTG in *B. sangzhiensis*, and ND2 using ATT, COI using GTG in *B. tuberogranulata*. Generally, stop codon usage patterns were similar between the two species but varied among different genes. In general, *B. sangzhiensis* employed six types of stop codons, while *B. tuberogranulata* used five. Both species shared TAG as stop codons for ND2, CYTB, and ND3; TAA for ATP8, ND4L, and ND5; the incomplete TA(A) for ND1, ATP6, and COIII; the incomplete T(AA) for COII and ND4; and AGA for COI. However, AGA was used as stop codons for ND6 in *B. tuberogranulata*, while that in *B. sangzhiensis* was AGG (Table 1). Nucleotide composition analysis of the 13 PCGs revealed similar negative AT and GC skew patterns, except for the ND6 gene, which exhibited an extraordinary but positive GC skew (Table 3).

**TABLE 3:** Nucleotide composition and skewness of different gene regions in the mitogenomes of *Boulenophrys sangzhiensis* (BS) and *Boulenophrys tuberogranulata* (BT).

	Length (bp)		T(U)%		C%		A%		G%		A + T%		AT skew		GC skew	
	BS	BT	BS	BT	BS	BT	BS	BT	BS	BT	BS	BT	BS	BT	BS	BT
ND1	974	974	34.39	34.19	28.64	28.85	23.31	23.72	13.66	13.24	57.70	57.91	−0.192	−0.181	−0.354	−0.371
ND2	1041	1041	31.41	30.55	31.12	31.51	26.32	26.99	11.14	10.95	57.73	57.54	−0.088	−0.062	−0.473	−0.484
COI	1557	1563	31.47	32.05	25.43	24.63	24.98	25.27	18.11	18.04	56.45	57.33	−0.115	−0.118	−0.168	−0.154
COII	685	685	29.49	31.68	27.74	24.96	27.15	27.74	15.62	15.62	56.64	59.42	−0.041	−0.066	−0.279	−0.230
ATP8	165	165	35.76	30.91	25.45	30.30	28.48	29.70	10.30	9.09	64.24	60.61	−0.113	−0.020	−0.424	−0.538
ATP6	683	683	33.82	31.77	28.99	29.43	24.74	26.94	12.45	11.86	58.57	58.71	−0.155	−0.082	−0.399	−0.426
COIII	785	785	28.92	32.36	29.81	27.52	24.59	24.46	16.69	15.67	53.50	56.82	−0.081	−0.139	−0.282	−0.274
ND3	345	345	32.75	35.65	30.72	27.25	22.03	22.61	14.49	14.49	54.78	58.26	−0.196	−0.224	−0.359	−0.306
ND4L	297	297	35.69	34.01	26.60	29.29	24.24	22.56	13.47	14.14	59.93	56.57	−0.191	−0.202	−0.328	−0.349
ND4	1378	1381	34.40	33.74	27.79	28.24	24.53	24.98	13.28	13.03	58.93	58.73	−0.167	−0.149	−0.353	−0.368
ND5	1818	1818	33.55	33.83	27.34	27.67	26.90	26.73	12.21	11.77	60.45	60.56	−0.110	−0.117	−0.382	−0.403
ND6	510	510	38.63	37.84	10.00	11.18	21.18	19.80	30.20	31.18	59.80	57.65	−0.292	−0.313	0.502	0.472
CYTB	1140	1140	33.16	31.67	28.60	29.91	24.12	24.30	14.12	14.12	57.28	55.96	−0.158	−0.132	−0.339	−0.359
PCG‐all	11,378	11,387	32.95	32.95	27.37	27.35	24.93	25.17	14.76	14.54	57.87	58.11	−0.139	−0.134	−0.299	−0.306
PCGs–^1st^	3795	3798	24.96	24.48	24.72	24.77	26.94	27.52	23.37	23.24	51.90	52.00	0.038	0.058	−0.028	−0.032
PCGs–^2nd^	3795	3798	41.49	41.55	27.58	27.39	18.19	18.35	12.74	12.71	59.68	59.90	−0.390	−0.387	−0.368	−0.366
PCGs–^3rd^	3788	3791	32.39	32.80	29.80	29.90	29.64	29.63	8.17	7.67	62.03	62.44	−0.044	−0.051	−0.570	−0.592
12S‐rRNA	925	925	22.92	23.24	26.38	26.38	30.16	30.38	20.54	20.00	53.08	53.62	0.136	0.133	−0.124	−0.138
16S‐rRNA	1594	1594	25.97	26.10	22.58	23.59	33.94	33.25	17.50	17.06	59.91	59.35	0.133	0.121	−0.127	−0.160
rRNAs	2519	2519	24.85	25.05	23.98	24.61	32.55	32.20	18.62	18.14	57.40	57.24	0.134	0.125	−0.126	−0.151
tRNAs	1529	1525	28.17	28.91	19.87	19.40	30.65	30.40	21.31	21.29	58.82	59.31	0.042	0.025	0.035	0.046
D‐loop	1496	1380	34.49	33.16	22.75	25.40	30.36	28.48	12.39	12.97	64.86	61.63	−0.064	−0.076	−0.295	−0.324

**FIGURE 3:**
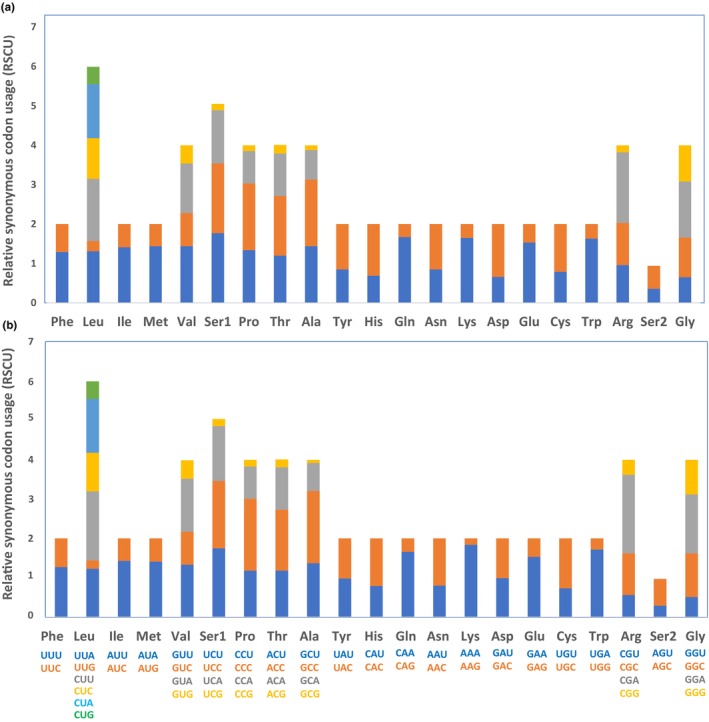
Relative synonymous codon usage (RSCU) of the mitogenome of *Boulenophrys sangzhiensis* (a) and *Boulenophrys tuberogranulata* (b).

Codon usage patterns of PCGs were similar in both *B. sangzhiensis* and *B. tuberogranulata* (Figure 3). Codons UCU (Ser1), UCC (Ser1), CCC (Pro), GCC (Ala), CAA (Gln), and CGA (Arg) had the highest frequencies in both species. The RSCU analysis indicated that Leu was encoded by six synonymous codons, while other amino acids were encoded by fewer: Val, Ser1, Pro, Thr, Ala, Arg, and Gly were encoded by four codons, and the remaining amino acids (Phe, Ile, Met, Tyr, His, Gln, Asn, Lys, Asp, Glu, Cys, Trp, and Ser2) were encoded by two codons (Figure 3).

### 3.3 Characteristics of rRNAs, tRNAs, and the control region

Both *B. sangzhiensis* and *B. tuberogranulata* had two rRNA genes: the 12S rRNA located between tRNA^Phe^ and tRNA^Val^ with a length of 925 bp in both species, and the 16S rRNA located between tRNA^Val^ and tRNA^Leu^ with lengths of 1594 bp in *B. sangzhiensis* and 1595 bp in *B. tuberogranulata*. Both mitogenomes contained 22 tRNAs, with total lengths of 1529 and 1525 bp for *B. sangzhiensis*, and *B. tuberogranulata*, respectively. Individual tRNA sizes ranged from 62 bp (tRNA^Ser1^) to 75 bp (tRNA^Leu2^) in *B. sangzhiensis* and from 63 bp (tRNA^Cys^) to 74 bp (tRNA^Leu2^) in *B. tuberogranulata*. Unlike the pattern of PCGs, both rRNAs and tRNAs had positive AT skew, and the tRNAs additionally exhibited positive GC skew, indicating a different base bias compared to PCGs. The noncoding control region, also known as the D‐loop, showed significant size variations, with a length of 1496 bp in *B. sangzhiensis* and 1380 bp in *B. tuberogranulata*. The D‐loop was located between tRNA^Pro^ and tRNA^Phe^ in both species, and it exhibited similar A + T contents, AT skew, and GC skew patterns with PCGs rather than rRNAs and tRNAs (Table 3).

### 3.4 Positive selection and phylogenetic relationships

**FIGURE 4:**
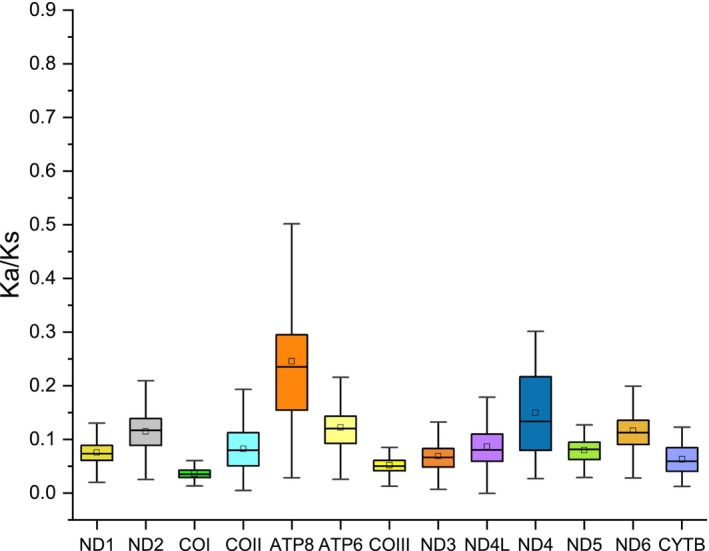
The Ka/Ks of 13 PCGs among 28 species within Megophryidae.

The Ka/Ks ratio was calculated to assess the evolutionary rates of each PCG (Figure 4). The highest Ka/Ks value was observed in ATP8 (0.246), while the lowest was in COI (0.037). Other PCGs such as ATP6, ND2, and ND4 exhibited relatively fast evolutionary rates, whereas COI, COIII, and CYTB showed relatively slow rates. However, all Ka/Ks ratios for the 13 PCGs were less than 1, indicating that they did not exhibit strong positive selection signals and possibly evolved under purifying selection.

**FIGURE 5:**
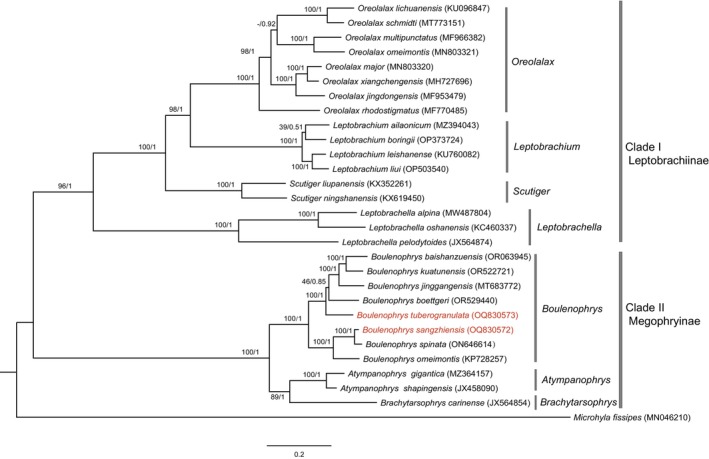
Phylogenetic relationships within Megophryidae derived from BI method based on 13 PCGs. The numbers on the branches represent the bootstrap values and posterior probabilities of ML/BI analyses. The number after species name is the GenBank accession number. Names in red show the phylogenetic positions of *Boulenophrys sangzhiensis* and *Boulenophrys tuberogranulata* that we sequenced in this study.

Both ML and BI analyses generated similar tree topologies (Figure 5). The Megophryidae family could be divided into two major clades, corresponding to the subfamilies Leptobrachiinae (Clade I) and Megophryinae (Clade II). Clade I could be further divided into four well‐supported major groups: *Leptobrachella*, *Scutiger*, *Oreolalax*, and *Leptobrachium*. Clade II contained species from the genera *Atympanophrys*, *Brachytarsophrys*, and *Boulenophrys*. Each examined genus appeared as monophyletic, and the intergeneric relationships among the two clades were also well‐supported. The genus *Boulenophrys* could be further divided into two subgroups, with the two species sequenced in this study appearing in each of these subgroups. *Boulenophrys sangzhiensis* grouped with *B. omeimontis* and then clustered with *B. spinata*. Comparatively, *B. tuberogranulata* clustered with *B. jinggangensis*, *B. boettgeri*, *B. kuatunensis*, and *B. baishanzuensis*.

## 4 DISCUSSION

Mitochondrial genomes (or called mitogenomes) serve as valuable molecular markers and have found widespread applications in molecular biology and ecology. In vertebrates, especially, mitogenomes are extensively utilized due to their conservative structure, typically comprising 13 PCGs, 2 rRNAs, 22 tRNAs, and 1 noncoding control region (D‐loop), with a sequence length usually ranging from 16 to 17 kb. In comparison to the complex nuclear DNA, vertebrate mitogenomes possess unique characteristics, including maternal inheritance, a rapid evolutionary rate, a simple structure with conserved coding regions, low levels of recombination, and multiple copy numbers (Boore, 1999; Zardoya & Meyer, 1996). These characteristics make mitochondrial DNA as valuable markers for reconstructing phylogenetic relationships, revealing population genetic structures, estimating divergence times, testing selective pressures, and identifying species using mitochondrial barcoding genes (Jiang et al., 2023; Lan et al., 2023; Shu et al., 2021; Zhang et al., 2023).

To the best of our knowledge, this study represents the first assembly of the mitogenomes for two horned toads, *B. sangzhiensis* and *B. tuberogranulata*. The exploration of similarities and differences in gene orders, genetic structures, base compositions, evolutionary features, and codon usage offers valuable molecular insights into the taxonomic and phylogenetic characteristics of closely related species (Sun et al., 2022). Overall, the mitogenomes of the two species exhibited similarities in terms of size, organization (Figure 2 and Table 1), nucleotide composition of PCGs, rRNAs, tRNAs, control region, and codon usage of PCGs (Figures 3 and 4 and Table 3). The most notable difference between the two mitogenomes was observed in the length of the D‐loop (1496 bp vs. 1380 bp), contributing significantly to the total length variations (16,950 bp vs. 16,841 bp). Despite this distinction, the two newly obtained mitogenomes exhibited patterns similar to those of other *Boulenophrys* species reported previously (Liu et al., 2016; Wu et al., 2024), such as negative AT skew and GC skew, along with high A + T content. The AT skew and GC skew generally reflect base bias, which can vary among different groups. For instance, the species we previously detected in Salamandridae had a positive AT skew (Wang, Lan et al., 2022), whereas all the Megophryidae species in this study displayed a negative AT skew, consistent with the findings of Zhou et al. (2023). Furthermore, while the mitogenomes of species in Ranoidea exhibited gene rearrangements frequently (Igawa et al., 2008), no any rearrangements were identified among all the Megophryidae species examined in this study.

By utilizing the mitogenomes of 26 species available from NCBI and the two newly obtained in this study as ingroups, with *M. fissipes* from the Microhylidae family as the outgroup, we successfully reconstructed a phylogenetic tree within Megophryidae based on the 13 PCGs of 29 species (Figure 5). The phylogenetic analysis revealed that Megophryidae could be divided into two well‐supported clades, supporting the classification of the two subfamilies, Megophryinae and Leptobrachiinae. The intergeneric relationships among the two subfamilies were consistent with our previous study (Zhou et al., 2023), with the monophyly of each examined genus also receiving support. Based on the two most recent phylogenetic studies of *Boulenophrys*, the genus can be further divided into three well‐supported species groups: the *B. minor* group, the *B. omeimontis* group, and the *B. boettgeri* group (Lyu et al., 2023; Qi et al., 2021). In this study, two species groups were identified and supported, although no sequences of the *B. minor* group were included. Specifically, *B. sangzhiensis*, *B. omeimontis*, and *B. spinata* formed the *B. omeimontis* group, while *B. tuberogranulata*, *B. jinggangensis*, *B. boettgeri*, *B. kuatunensis*, and *B. baishanzuensis* grouped in the *B. boettgeri* group. These phylogenetic relationships within *Boulenophrys*, as elucidated here using mitogenomes, were largely consistent with previous studies that employed more species but fewer molecular markers (Lyu et al., 2023; Qi et al., 2021).

Interestingly, despite *B. sangzhiensis* and *B. tuberogranulata* being sympatric and coexisting in Zhangjiajie City, Hunan Province, this study revealed that they belong to two distinct phylogenetic clades (Figure 5). *Boulenophrys sangzhiensis*, along with other species in the *B. omeimontis* species group, mainly displayed a southwestern distribution pattern. In contrast, *B. tuberogranulata* and other species in the *B. boettgeri* species group were primarily distributed in the southeastern area of China. It appears that Zhangjiajie City serves as the boundary area between the two clades of *Boulenophrys*, especially considering that the type localities of *B. sangzhiensis* and *B. tuberogranulata* completely overlapped in Mt. Tianping in Sangzhi County. A similar pattern was observed in mustache toads of *Leptobrachium* we studied before, where *L. boringii* and *L. liui* were also collected in the same region of Zhangjiajie City but occupied very different phylogenetic lineages that showed a parallel southwestern and southeastern convergence (Zhou et al., 2023). Notably, another species, *B. caudoprocta*, was also found in Mt. Tianping in the same area (Figure 1). Although the phylogenetic position of *B. caudoprocta* was not assessed in this study, a previous investigation indicated that the three species found in Mt. Tianping did not form any sister group relationships and they were also morphologically distinct (Lyu et al., 2023). This distribution pattern in *Boulenophrys* is unique, with three species coexisting in the same mountain but having distinct phylogenetic histories. This pattern highlights the special role of the coexisting area, the Zhangjiajie City in driving the speciation of these species. Similar coexistence of species in nearby areas was also observed in other species of *Boulenophrys*. For instance, the type localities of *B. boettgeri*, *B. ombrophila* and *B. kuatunensis* were all in Kuatun village (Fujian Province, China), *B. yangmingensis* and *B. xiangnanensis* were both in Mt. Yangming (Hunan province, China), and *B. cheni*, *B. jinggangensis*, and *B. lini* were all found in Mt. Jinggang (Jiangxi Province, China). However, these sympatric species were all clustered in the same phylogenetic clade (Lyu et al., 2023).

There are more than 300 species in Megophryidae; however, the available mitogenome data are still very limited. As of now, only 25 complete and nine nearly complete mitogenomes belonging to 26 recognized species of Megophryidae are available from NCBI. The phylogenetic tree presented in this study, based on 28 mitogenomes in Megophryidae, demonstrated that intergeneric and intrageneric relationships can be reliably elucidated with high supporting values. It suggests that mitogenomes serve as valuable molecular markers for constructing a robust phylogenetic tree of Megophryidae. It is worth noting that the advent of NGS has revolutionized genomics research by enabling the sequencing of entire genomes with ever‐increasing throughput and decreasing costs (Van Dijk et al., 2014). With the application of NGS, we anticipate that the complete evolutionary history of Megophryidae will gradually be unveiled when more mitogenomes, such as the two *Boulenophrys* species in this study, become available in the future.

## AUTHOR CONTRIBUTIONS

**Hongmei Xiang:** Conceptualization (equal); data curation (lead); formal analysis (equal); investigation (equal); methodology (equal); software (lead); visualization (lead); writing – original draft (lead); writing – review and editing (equal). **Qiang Zhou:** Investigation (equal); methodology (equal); visualization (supporting); writing – original draft (supporting); writing – review and editing (supporting). **Wei Li:** Formal analysis (supporting); investigation (equal); resources (equal); writing – review and editing (supporting). **Juan Shu:** Formal analysis (supporting); investigation (equal); resources (equal); writing – review and editing (supporting). **Zhirong Gu:** Formal analysis (supporting); investigation (equal); resources (equal); writing – review and editing (supporting). **Wansheng Jiang:** Conceptualization (lead); data curation (equal); formal analysis (equal); funding acquisition (lead); investigation (equal); methodology (supporting); project administration (lead); software (supporting); visualization (supporting); writing – review and editing (lead).

## CONFLICT OF INTEREST STATEMENT

The authors declare no conflicts of interest.

## Data Availability

The final complete mitogenomes, along with annotated information for both species, have been deposited under GenBank accession numbers OQ830572 (*B. sangzhiensis*) and OQ830573 (*B. tuberogranulata*).
